# Stress Degradation Behavior of Abacavir Sulfate and Development of a Suitable Stability-Indicating UHPLC Method for the Determination of Abacavir, its Related Substances, and Degradation Products

**DOI:** 10.3797/scipharm.1206-11

**Published:** 2012-07-27

**Authors:** Pallavi Vukkum, Girish R. Deshpande, J. Moses Babu, R. Muralikrishna, Pavani Jagu

**Affiliations:** 1 Analytical Research, Custom Pharmaceutical Services, Dr. Reddy’s Laboratories Ltd., Bollaram road, Miyapur, Hyderabad-500049 (AP), India.; 2 Department of Chemistry, Andhra University, Visakhapatnam-530003, India.

**Keywords:** Abacavir Sulfate, UHPLC, Forced degradation, Identification, Validation, LC-MS

## Abstract

A novel, stability-indicating UHPLC method was developed for the quantitative determination of Abacavir sulfate, its related substances, and forced degradation impurities in bulk drugs. The chromatographic separation was achieved on a Waters Acquity BEH C_8_, 50 mm × 2.1 mm, 1.7 μm particle size column with a mobile containing a gradient mixture of solution A (0.10 % v/v *o*-phosphoric acid in water) and solution B (0.10% v/v *o*-phosphoric acid in methanol). The flow rate was set at 0.40 mL/min and the run time was 6.0 min. The drug substance was subjected to the stress studies of hydrolysis, oxidation, photolysis, and thermal degradation. Abacavir sulfate was found to degrade significantly under acidic hydrolysis and oxidative stress conditions. The formed degradation products were reported and were well-resolved from Abacavir and its related substances. The mass balance was found to be satisfactory in all of the stress conditions, thus proving the stability-indicating capability of the method. The developed UHPLC method was validated to be in agreement with ICH requirements and found to be rapid, accurate, precise, linear, specific, and suitable for the quantitative determination of related substances and degradants in the bulk drug samples of Abacavir sulfate.

## Introduction

Abacavir Sulfate, {(1*S*,4*R*)-4-[2-amino-6-(cyclopropylamino)-9*H*-purin-9-yl]cyclopent-2-en-1-yl}methanol sulfate (2:1), is a nucleoside reverse transcriptase inhibitor (NRTIs) [1]. It is used either as a 600-mg once-daily or 300-mg twice-daily regimen exclusively in the treatment of human immunodeficiency virus (HIV) infection [2], and mainly helps to halt the inroads of the human immunodeficiency virus (HIV). Without treatment, HIV gradually undermines the body's immune system, encouraging other infections to take hold until the body succumbs to full-blown acquired immune deficiency syndrome (AIDS). Initially, Abacavir is phosphorylated to its corresponding monophosphate as an intracellular reaction. Cytosolic enzymes convert Abacavir monophosphate to carbovir monophosphate (CBV-MP), which is finally phosphorylated to the biologically active moiety, carbovir triphosphate (CBV-TP). CBV-TP inhibits HIV reverse transcriptase by competing with the endogenous substrate dGTP and by chain termination subsequent to incorporation into the growing polynucleotide strand [3].

With no end in sight to the worldwide shortage of solvents, power, time, and many valuable resources, pharmaceutical laboratories are in search of cost-effective solutions to manage this impact on their research and business timeline. Recently, commercially available ultra-high performance liquid chromatography (UHPLC) has been proven to be one of the most promising developments in the area of fast chromatographic separations by reducing analysis time and maintaining high efficiency without compromising quality. Many HPLC methods have been reported in the literature for the determination of Abacavir in plasma and therapeutic monitoring, and simultaneous determination with other antiretroviral products [4–11], pharmaceutical dosage forms and human serum [12], in biological matrices [13, 14], and electrochemical determination [15]. Though HPLC is a well-established, reliable technique used in controlling the quality and consistency of active pharmaceutical ingredients (API's) and dosage forms, it is often a slow technique because of the complexity of some of the samples, and it could still be improved. Ultra-high performance liquid chromatography (UHPLC) is a new category of separation techniques based upon well-established principles of liquid chromatography, which utilizes sub-2 μm particles for the stationary phase. These particles operate at elevated mobile phase linear velocities to affect dramatic increases in resolution, sensitivity, and speed of analysis [16]. Because of its speed and sensitivity, this technique is gaining considerable attention in recent years for pharmaceutical and biomedical analysis [17–20]. In the present work, this technology has been applied to the method development and validation study of related substances and the assay determination of Abacavir sulfate bulk drug.

To the best of our knowledge, no stability-indicating UHPLC method for the quantitative estimation of Abacavir sulfate in drug substances and in pharmaceutical dosage forms has been reported. The present research work is mainly focused to develop a rapid, sensitive, and accurate UHPLC method for the determination of Abacavir sulfate, its related substances, and degradants. The developed method was validated with respect to specificity, LOD, LOQ, linearity, precision, accuracy, and robustness. Forced degradation studies were performed on the drug substance to show the stability-indicating capability of the method. All of these studies were performed in accordance with established ICH guidelines. In the present work, an attempt was made to show how UHPLC can reduce analysis time with fewer amounts of solvents, but without compromising the resolution and sensitivity.

## Experimental

### Compounds

Samples of Abacavir sulfate and its potential impurities were synthesized by the process research department of Custom Pharmaceutical Services, Dr. Reddy's Laboratories, Hyderabad, India. HPLC grade methanol, analytical grade *o*-phosphoric acid, sodium hydroxide, hydrochloric acid, and hydrogen peroxide were purchased from Rankem (Mumbai, India). High-purity water was prepared using a Millipore Milli-Q plus (Millipore, Milford, MA, USA) purification system.

### Instrumentation

The UHPLC system (UFLC_XR_ from Shimadzu, Kyoto, Japan) consisted of two LC-20AD pumps, the SPD-M20A diode array detector, SIL-20AC auto sampler, and the LC-20A3 degasser. A reverse phase Acquity BEH C_8_ with the dimensions 50 mm in length, 2.1 mm in ID, and 1.7 μm particle size (Waters CORP, Milford, USA) was used for achieving the separation of all the compounds. The chromatographic data was recorded using an HP-Vectra (Hewlett-Packard, Waldron, Germany) computer system with LC solutions data acquiring software (Shimadzu, Kyoto, Japan). LC-MS was performed using the Agilent 1100 series liquid chromatography system coupled with the 6410-series triple quadruple mass spectrometer. The Cintex digital water bath was used for the hydrolysis studies. The photostability study was carried out in a photostability chamber (Sanyo, Leicestershire, UK). The thermal stability study was carried out in a dry air oven (Cintex, Mumbai, India).

### Chromatographic conditions

A simple, rapid UHPLC method was developed for the determination of Abacavir sulfate, its related substances, and degradation compounds. Chromatographic separations were achieved on a Acquity BEH C_8_ (50 mm × 2.1 mm ID, 1.7 μm) column having a stationary phase of ethylene-bridged hybrid octylsilanes, and a mobile phase system containing 0.10% v/v *o*-phosphoric acid in water (Mobile phase A) and 0.10% v/v *o*-phosphoric acid in methanol (Mobile phase B), by employing a gradient program (T/ %B) = 0/8, 5/40, 6/40, 6.01/8 with a flow rate of 0.40 mL/min. The column temperature was maintained at 40° C and the detection wavelength was set to 220 nm. The injection volume was 10 μL, with a sample loading of 0.001 mg. Water was used as the diluent. The mobile phase was filtered through a 0.45μm PTFE filter (Millipore, USA) and degassed by sonication just before use.

### LC–MS conditions

The LC–MS system (Agilent 1100 series liquid chromatography system coupled with a 6410 series triple quadruple mass spectrometer) was used for the identification of unknown compounds formed during forced degradation. Identification was achieved on a Acquity BEH C_8_ (50 mm×2.1 mm i.d., 1.7 μm) column, with the mobile phase system containing 0.10% v/v formic acid in water (Mobile phase A) and 0.10% v/v formic acid in methanol (Mobile phase B), by employing a gradient program as (T/%B) = 0/8, 5/40, 12/40, 12.01/8 with a flow rate of 0.40 mL/min. The column temperature was maintained at 40° C and the detection wavelength was set to 220 nm. The analysis was performed in both positive and negative electrospray ionization modes. The capillary voltage was 4.0 kV. The source temperature was 300°C and the gas flow rate was 600 L/h.

### Preparation of standard solutions and sample solutions

The standard solution of Abacavir sulfate was prepared at a concentration of 0.10 mg/mL by dissolving an appropriate amount of the drug substance in water. Stock solutions of each impurity were prepared at a concentration of 0.10 mg/mL in a water: acetonitrile mixture (1:1 v/v). The analyte concentration of the Abacavir sulfate test sample was fixed at 0.10 mg/mL. The standard solutions were prepared by diluting the stock solutions to attain the required concentration of impurities and drug substance with water diluent.

### Stress studies/specificity

Specificity is the ability to unequivocally assess the analyte in the presence of its potential impurities [21], which may be expected to be present like impurities, degradants, matrix, etc. [22]. The specificity of the developed UHPLC method for Abacavir sulfate was established in the presence of its known impurities, namely Imp-A, Imp-B, Imp-C, Imp-D, Imp-E, Imp-F, Imp-G, and its degradation products. The ability of the method to separate all of the compounds was assessed by evaluating the resolution between the peaks corresponding to the various compounds to show the stability-indicating ability and specificity of the proposed UHPLC method.

**Fig. 1.**
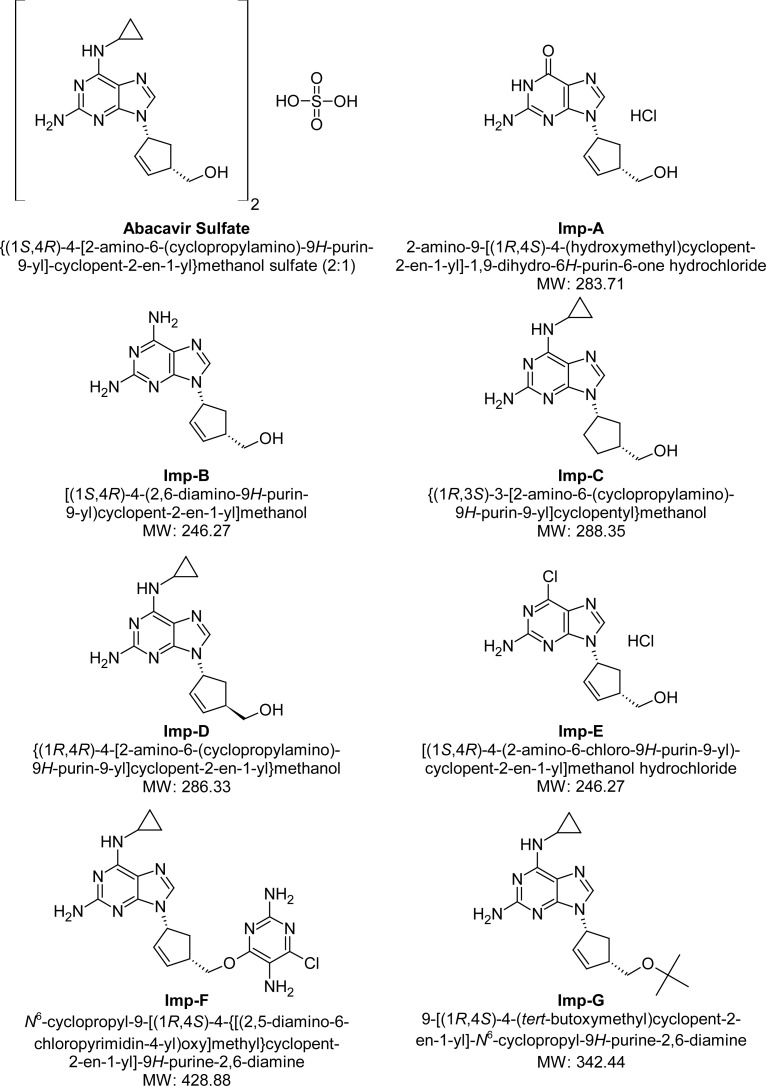

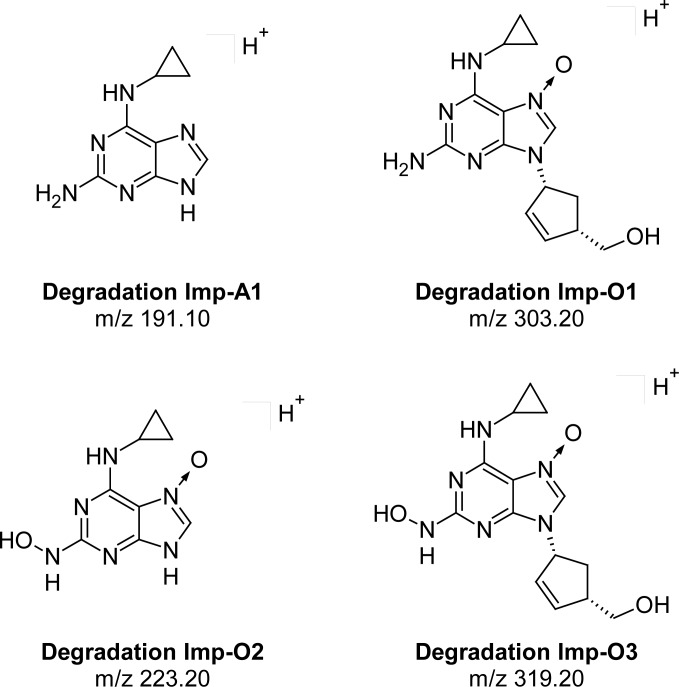
Structures of Abacavir sulfate, its related impurities, and forced degradation impurities

The stress conditions employed for the degradation studies as per ICH recommendations include photolytic, thermal, oxidation, and hydrolysis with acid and base. The photolytic stress study was performed for 11 days at 200 W h/ m^2^ of UV light and 1.2 million lux hours of visible light. The thermal stress study was performed at 105°C for 10 days. The acid and base stress studies were performed with 1 N HCI and 1 N NaOH for 42 h at ambient temperature (25 ± 2°C). The oxidation stress was done with 3% H_2_O_2_ solution for seven days at ambient temperature. All of the stressed samples were quantified against the Abacavir reference standard. The purity of the Abacavir peak in the stressed samples and the spiked solution of Abacavir sulfate with its known related impurities was checked by a photodiode array detector (PDA). The structures of Abacavir sulfate, its related impurities, and degradation products are shown in Fig. 1.

### Method Validation

The described UHPLC method has been extensively validated for the assay and related substances as per ICH guidelines [23].

### System suitability test

The system suitability test is an integral part of the chromatographic methods and is used to verify that the resolution and reproducibility of the chromatographic system are adequate for the analysis to be performed. The system suitability test results of the UHPLC method on the Acquity BEH C_8_ column are computed in Table 1.

### Limit of detection (LOD) and limit of quantification (LOQ)

The LOD and LOQ of Abacavir sulfate and its related impurities were determined by diluting their known concentrations that would yield a signal-to-noise ratio of 3:1 and 10:1, respectively. Precision was carried out at the LOQ level by preparing six individual preparations of Abacavir sulfate with its related impurities at the LOQ level, and calculating the percentage RSD for the areas of Abacavir and its related impurities. Accuracy at the LOQ level was also carried out by preparing three recovery solutions of Abacavir with its related impurities at the LOQ level, and calculating the percentage recovery for areas of all related impurities.

### Linearity

The linearity of the method was established at two different levels. The assay linearity was performed by preparing five different solutions like 50, 75, 100, 125, and 150% w/w with respect to the target analyte concentration of Abacavir sulfate. The linearity at a low level was performed by preparing six different solutions corresponding to the LOQ, 0.05, 0.075, 0.10, 0.15, and 0.20% w/w of Imp-A, Imp-B, Imp-C, Imp-D, Imp-E, Imp-F, Imp-G, and Abacavir sulfate with respect to the analyte concentration. The peak area versus concentration data was plotted for linear regression analysis. The correlation coefficients of regression, slope, intercept, and percent y-intercept of the calibration curves were computed. The relative response factor (RRF) of each impurity was determined by dividing the slope of each impurity by the slope of Abacavir sulfate.

### Precision

Six individual measurements of Abacavir sulfate were performed with 0.10 % w/w of each of Imp-A, Imp-B, Imp-C, Imp-D, Imp-E, Imp-F, and Imp-G with respect to the target analyte concentration. The assay and the content of each impurity were determined for each of the preparations using RRF values wherever applicable. The method’s precision was evaluated by calculating the percentage RSD of assay values and impurity content in six preparations. Experiments with a different analyst, column, and instrument in the same laboratory were performed in order to ascertain the intermediate precision or ruggedness of the developed method.

### Accuracy

The accuracy of the assay was evaluated in triplicate at three concentration levels, i.e. 50, 100, and 150 μg/mL of Abacavir sulfate. The percentage recovery at each level was calculated. Standard addition and recovery experiments were conducted to determine the accuracy of the related substance in the same method for the quantification of all seven impurities in Abacavir sulfate. The study was carried out in triplicate at 0.05, 0.10, and 0.15% w/w of the target specification limit. The percentage recoveries for Imp-A, Imp-B, Imp-C, Imp-D, Imp-E, Imp-F, and Imp-G were calculated considering the amount of impurity-spiked, the amount of impurity available in the un-spiked sample, and the amount of impurity recovered after the RRF correction.

### Robustness

The robustness of an analytical procedure is a measure of its capacity to remain unaffected by small, but deliberate variations in method parameters, and provides an indication of its reliability during normal usage. To determine the robustness of the developed UHPLC method, deliberate changes were made from original experimental conditions. The effect of the flow rate was studied at 0.3 mL/min and 0.5 mL/min, instead of at 0.4 mL/min. The effect of wave length was studied at 218 nm and 222 nm, instead of at 220 nm. The effect of the column temperature was studied at 35°C and 45°C, instead of at 40°C. The effect of the gradient program was studied with the program (T/%B): 0/6, 5/38, 6/38, 6.01/6, and (T /%B): 0/10, 5/42, 6/42, 6.01/10, instead of with (T /%B): 0/8, 5/40, 6/40, 6.01/8. For all of the changed conditions i.e. flow rate, wavelength, temperature, and gradient program, the chromatographic parameters were computed like theoretical plates, the tailing factor of the analyte, and the resolution between the critical pair i.e. between the analyte and Imp-C.

### Solution stability and mobile phase stability

The solution stability of Abacavir sulfate in the presence of its related impurities at the specification level was carried out by keeping the solution in a tightly capped volumetric flask at room temperature (25±2°C) on a laboratory benchtop for 48 h. The contents of all related impurities were determined at 6 h intervals up until the study period of 48 h.

The mobile phase stability was carried out by evaluating the content of all related impurities in the Abacavir sulfate sample solution, which was spiked with known impurities at the specification level, prepared freshly at each 6 h interval up until 48 h, while the same mobile phase was used during the study period.

## Results and Discussion

### Method development and optimization

Imp-A, Imp-B, Imp-C, Imp-D, Imp-E, Imp-F, and Imp-G are the potential impurities of the Abacavir sulfate drug substance. The main objective of the chromatographic method was to separate all potential impurities and the degradation products from the analyte. Imp-C was co-eluted with the analyte peak while using different stationary phases, such as C18, cyano, and phenyl, and with various mobile phases, such as trifluoroacetic acid and acetate buffers, and organic modifiers including acetonitrile and methanol. Selection of the column has played a critical role in achieving the separation of Imp-C from the analyte peak. Development of the method was initiated by using 0.05% trifluoroacetic acid in water as the mobile phase A and 0.05% trifluoroacetic acid in acetonitrile as the mobile phase B with a gradient program (T/%B): 0/5, 5/50, 6/50, 6.01/5 post-run: 5 min at a flow rate of 0.40 mL/min. The column used was 50 mm in length, 2.1 mm internal diameter, and 1.7 μm particle size C18 stationary phase. When a solution spiked with impurities was injected into the UHPLC system, the system suitability parameters for the analyte peak were observed to be very poor with very little resolution between the Imp-C and analyte peak. Imp-C almost co-eluted with the analyte, hence the peak purity of the analyte also failed. To improve the peak shape, mobile phase A was replaced with a 10 mM ammonium acetate buffer solution and the spiked solution was injected into the UHPLC system. The peak shape was slightly improved, but the Imp-C still showed little resolution from the analyte peak.

The next trial was done with 0.10 % *o*-phosphoric acid in water as mobile phase A and 0.10% *o*-phosphoric acid in methanol as mobile phase B. After injecting the spiked solution, the analyte peak was seen to be improved in theoretical plates and the tailing factor, but the Imp-C still had lower resolution from the analyte peak. The C-18 stationary phase was not successful in achieving a satisfactory resolution of the Imp-C and analyte peaks, and in avoiding co-elution of other process impurities. The next trials were performed on cyano and phenyl columns but results were not satisfactory, so the C-8 stationary phase was employed with 0.10% *o*-phosphoric acid in water as mobile phase A and 0.10% *o*-phosphoric acid in methanol as mobile phase B, and a different gradient program.

**Fig. 2.**
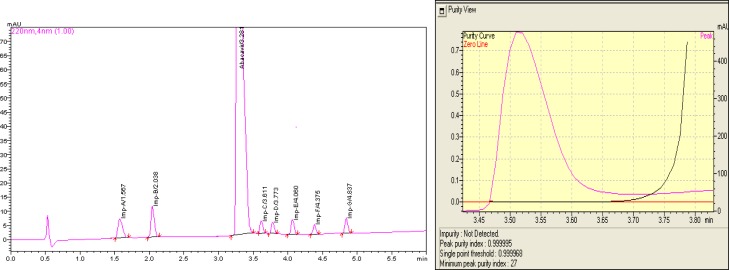
Typical UHPLC chromatogram and peak purity spectrum of Abacavir sulfate spiked with its related impurities

Chromatographic separation was successfully achieved on the C_8_ stationary phase, Acquity BEH C_8_ (50 mm length, 2.1 mm internal diameter, 1.7 μm particle size) column having a stationary phase of ethylene-bridged hybrid octylsilanes, and the mobile phase system contained 0.10% *o*-phosphoric acid in water (mobile phase A) and 0.10% *o*-phosphoric acid in methanol (mobile phase B). The gradient program was modified to (T/%B): 0/8, 5/40, 6/40, 6.01/8, at a flow rate of 0.4 mL/min. The column temperature was maintained at 40° C and the detection wavelength was set at 220 nm. The injection volume was 10 μL, with a sample loading of 0.001 mg. In the optimized conditions, the impurities, namely Imp-A, Imp-B, Imp-C, Imp-D, Imp-E, Imp-F, Imp-G, and drug substance Abacavir sulfate, were well-separated with a resolution of more than 3.0, and all of thedegradation impurities were separated with a resolution of more than 2.0 from the analyte peak. The typical chromatogram of Abacavir sulfate with its related impurities is presented in Fig. 2. The method was specific to separate Abacavir sulfate from its potential impurities and degradant impurities. The system suitability test results are shown in Table 1.

### Results from Method Validation

#### Results of forced degradation studies

**Fig. 3.**
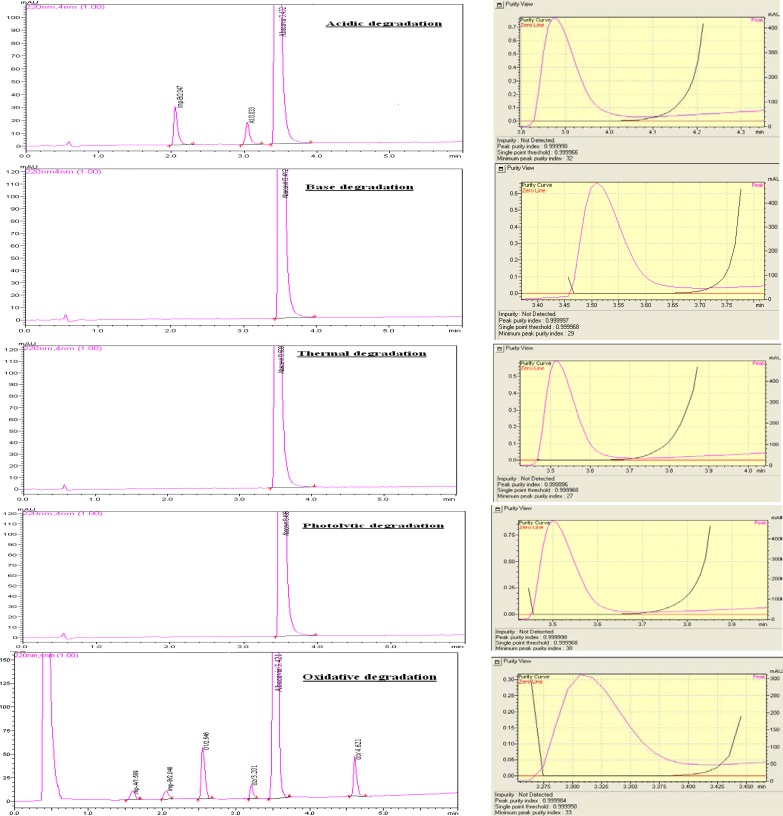
Specificity in presence of degradation products

No degradation was observed in the stressed conditions when the analyte was subjected to photolytic, thermal, and base hydrolysis. The degradation of the drug substance was observed under acidic and oxidative conditions. The chromatograms are presented in Fig. 3. Abacavir sulfate under acid hydrolysis led to the formation of an unknown degradation Imp-A1 at RRT 0.88 along with known Imp-B. Abacavir sulfate under oxidative conditions led to the formation of unknown degradation impurities, named Imp-O1, Imp-O2, and Imp-O3 at RRT 0.74, 0.94, and 1.35, respectively, along with known impurities Imp-A & Imp-B. The peak purity index over the single point threshold obtained in all stressed samples for the analyte peak, demonstrates the specificity of the UHPLC method. The assay of Abacavir sulfate is unaffected in the presence of Imp-A, Imp-B, Imp-C, Imp-D, Imp-E, Imp-F, Imp-G, and its degradation products. The mass balance in all stressed samples was nearly 99.0 % w/w, when the RRF of the degradant impurities was considered to be one. All of the studies confirm the specificity and stability-indicating ability of the developed UHPLC method. The summary of the forced degradation is captured in Table 2 and Table 3.

### Identification of major degradation products formed in acidic and oxidative degradations

**Fig. 4.**
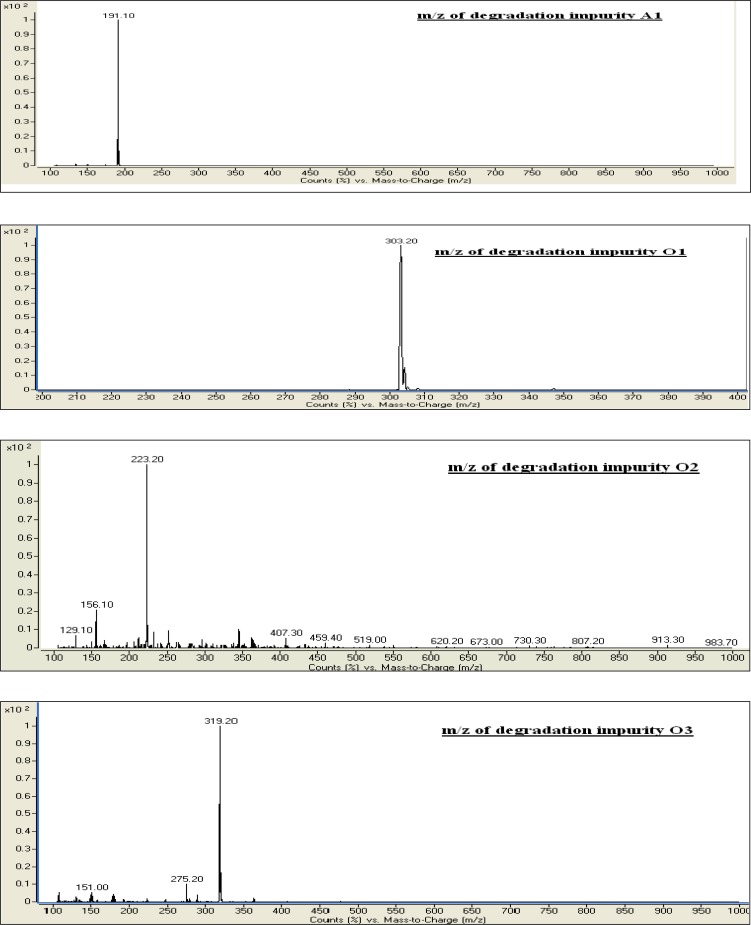
Typical mass spectra of degradation products

A LC–MS study was carried out to determine the m/z value of the major degradation products formed during acidic hydrolysis and oxidative degradation using an Agilent 1100 series liquid chromatography system coupled with a 6410 series triple quadruple mass spectrometer. The LC-MS conditions were already described in Section 2.4. The m/z values obtained for the unknown degradation products Imp-A1, Imp-O1, Imp-O2, and Imp-O3 were 191.10, 303.20, 223.20, and 319.20, respectively, and corresponds to the molecular weights 190.10, 302.20, 222.20, and 318.20, respectively [24]. The proposed structures for the degradation impurities are shown in Fig. 1 and the typical mass spectra of the degradation products are shown in Fig. 4.

### Precision

The precision of an analytical procedure expresses the closeness of agreement among a series of measurements obtained from multiple samplings of the same homogenous sample under prescribed conditions. The %RSD of the assay results of Abacavir sulfate during the method precision study was less than 0.3 % and the %RSD of the content of Imp-A, Imp-B, Imp-C, Imp-D, Imp-E, Imp-F, and Imp-G were within 3.8 %. The %RSD of the assay results obtained in the intermediate precision study was 0.4 %, and the %RSD contents of Imp-A, Imp-B, Imp-C, Imp-D, Imp-E, Imp-F, and Imp-G were less than 4.8%. Also, the individual values fall well within the range of the confidence interval of the average, confirming the precision of the method. The %RSD values are reported in Table 4 & Table 5 for the related impurities and Abacavir sulfate, respectively.

### Limit of Detection (LOD) and Limit of Quantitation (LOQ)

**Fig. 5.**
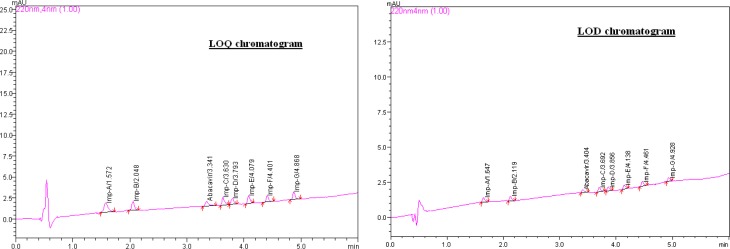
Typical LOQ and LOD chromatograms of Abacavir sulfate with its related impurities

The LODs of Abacavir sulfate, Imp-A, Imp-B, Imp-C, Imp-D, Imp-E, Imp-F, and Imp-G were less than or equal to 0.02% w/w (with respect to the target analyte concentration) for the 10μL injection volume (Fig. 5). The LOQs of Abacavir sulfate, Imp-A, Imp-B, Imp-C, Imp-D, Imp-E, Imp-F, and Imp-G were less than or equal to 0.04% w/w (of the target analyte concentration) for the 5μL injection volume (Fig. 5). The RSDs of the impurity content at the LOQ level were less than 2.0 % and the recovery values at the LOQ level were from 94.1 to 103.3. The LOD & LOQ values of Abacavir sulfate and its related impurities, and the precision at the LOQ level are tabulated in Table 4. The accuracy results at the LOQ level are tabulated in Table 6.

### Linearity

The linear calibration plot for the assay was obtained over the calibration ranges tested, i.e. 50–150 μg/mL and the correlation coefficient obtained was greater than 0.999. The values of the slope, intercept, and %Y-intercept of the calibration curves were determined. An excellent correlation existed between the peak area and the concentration of Abacavir sulfate for the assay determination. The results are computed in Table 5.

The linear calibration plot for the related substances was obtained over the calibration ranges tested, i.e. the LOQ-0.20% w/w for Abacavir sulfate, Imp-A, Imp-B, Imp-C, Imp-D, Imp-E, Imp-F, and Imp-G. The correlation coefficients obtained for all of the impurities were also greater than 0.999. The values of the slope, intercept, and %Y-intercept of the calibration curves were determined. The RRF of each impurity was determined using the slope of each impurity plot against the Abacavir sulfate plot. The Y-intercept of each plot was within 3.0% of the response at the 0.10% w/w level of each impurity, depicting that the plot almost crosses the origin. This enables an exact value of the RRF to be obtained, which will minimize the error in the quantification of impurities. The linearity results and RRF values are computed in Table 4.

### Accuracy

The percentage recovery of Imp-A, Imp-B, Imp-C, Imp-D, Imp-E, Imp-F, and Imp-G in the bulk drug samples ranged from 94.1 to 105.0 (Table 6). The percentage recovery of Abacavir sulfate in the bulk drug samples ranged from 99.3 to 100.1 (Table 5). All of the individual recovery values of the assay and impurities were well within the confidence interval of the mean values. Good recovery values were obtained, reflecting the exact values of the RRF for all of the impurities as well as the capability of the method’s accuracy.

### Robustness

In all of the deliberate varied chromatographic conditions carried out as described above (flow rate, mobile phase ratio by gradient change, wave length, and column temperature), the tailing factor of Abacavir sulfate is less than 1.1, the theoretical plates are more than 65,000, and the resolution between Abacavir sulfate and Imp-C is greater than 2.4. A very minor variation in the theoretical plates, resolution, and tailing factor results observed in all the robustness conditions illustrated the robustness of the method. Though the higher column temperature showed a better tailing factor, it is preferable to run in a nominal temperature when considering the durability of the column. The results are shown in Table 7.

### Solution stability and mobile phase stability

The %RSD of the assay of Abacavir sulfate during the solution stability and mobile phase stability experiments were within a 0.80 % variation. No significant changes were experienced in the content of any of the impurities during the solution stability and mobile phase stability experiments. The accuracy of the assay at each time point against the initial value is between 99.5 and 100.6%. The accuracy of the content of each impurity against the initial value is between 96.4 and 103.3%. The solution stability and mobile phase stability experimental data confirmed that the sample solutions and mobile phase used were stable up to 48 h. It is an advantage that from the same run, the assay results and impurities’ quantification can be derived. This helps to reduce the analysis time and the number of samples that can be analyzed until 48 h in the same sequence in the quality control during the regular analysis.

## Conclusion

The developed simple UHPLC method for the related substance and assay determination of Abacavir sulfate is linear, precise, accurate, and specific. The short run time for the analytical method saves a lot of analysis time (∼6 times faster) as well as the cost of the solvents to be used, which is reduced significantly (∼3 times lower). The method was validated to the requirements of ICH and the results were satisfactory. The developed stability-indicating UHPLC method can be used for the routine analysis of production samples and also to check the stability of bulk samples of Abacavir sulfate during its storage.

Communication number IPDO IPM – 00275 has been allotted for this research article in the research laboratory.

## Figures and Tables

**Tab. 1.** Results of system suitability test

**Cpd.**	**RT(min)**	**RRT^a^ (n=3)^c^**	**USP resolution^b^ (n=3)^c^**	**USP tailing factor (T_f_) (n=3)^c^**	**No. of theoretical plates (USP tangent method)**
Imp-A	1.6	0.48±0.01	–	1.2±0.03	38,042
Imp-B	2.0	0.61±0.01	3.7±0.51	1.1±0.02	42,299
Abacavir Sulfate	3.3	1.00±0.00	9.9±0.75	1.1±0.03	86,456
Imp-C	3.6	1.09±0.01	3.4±0.26	1.1±0.01	193,784
Imp-D	3.8	1.15±0.01	2.1±0.39	1.1±0.02	173,907
Imp-E	4.1	1.24±0.01	3.3±0.68	1.2±0.02	187,758
Imp-F	4.4	1.33±0.01	4.1±0.71	1.0±0.04	247,721
Imp-G	4.8	1.45±0.02	5.8±0.96	1.1±0.03	340,097

a Relative retention times (RRT) were calculated against the retention time (RT) of Abacavir sulfate;

b Resolution calculated between two adjacent peaks;

c Mean ± SD.

**Tab. 2.** Summary of forced degradation results

**Stress condition**	**Time**	**Analyte after degradation**		**Remarks**
		**Purity**	**Assay**	
Unstressed sample	–	99.8	99.6	–
Acid hydrolysis (1N HCl)	42 h	92.1	92.7	Unknown degradation impurity A1 along with known impurity-B were formed
Base hydrolysis (1N NaOH)	42 h	99.5	99.4	No degradation products formed
Oxidation (3% H_2_O_2_)	7 d	91.3	91.9	Unknown degradation impurities O1, O2, O3 along with known impurities-A & B were formed
Thermal (105° C)	10 d	99.7	99.5	No degradation products formed
Photolytic degradation as per ICH guidelines	11 d	99.6	99.4	No degradation products formed

**Tab. 3.** Results of degradation studies

**Degradation Impurity**	**Mass number (m/z)**	**Relative retention time (RR_t_) min**	**Tailing factor (T_f_)**
A1	191.10	0.88	1.0
O1	303.20	0.74	1.0
O2	223.20	0.94	1.1
O3	319.20	1.35	1.0

**Tab. 4.** Results of validation parameters for related impurities

**Parameter**	**Abacavir**	**Imp-A**	**Imp-B**	**Imp-C**	**Imp-D**	**Imp-E**	**Imp-F**	**Imp-G**
LOD (mg/mL)	0.008	0.015	0.008	0.007	0.005	0.006	0.008	0.008
LOQ (mg/mL)	0.023	0.044	0.024	0.020	0.015	0.018	0.024	0.023
Linearity^a^								
Slope (m)	21531.1	9817.5	21700.7	21709.4	22704.7	25807.1	20634.7	20340.8
Intercept (C)	−13.09	−33.29	−19.8	2.41	−55.90	11.44	3.2	−7.51
% Y-intercept	−0.35	−0.83	−0.58	0.07	−2.20	0.32	0.09	−0.21
Correl. coefficient	0.9999	0.9994	0.9999	0.9995	0.9997	0.9999	0.9994	0.9992
Precision at LOQ level (%RSD)^b^	1.42	0.74	0.81	1.88	0.91	1.02	1.14	1.98
Precision (%RSD)^b^	–	3.31	2.42	3.12	3.76	1.87	3.28	3.57
Ruggedness(%RSD)^b^	–	3.95	3.91	4.77	3.11	3.82	1.61	3.54
Relative response factor	1.00	0.46	1.01	1.01	1.06	1.20	0.96	0.95

a Linearity range was from LOQ to 0.20 %w/w of Abacavir sulfate and its related impurities with respect to analyte concentration;

b (n=6).

**Tab. 5.** Results of validation parameters for Abacavir sulfate at assay level

**Parameter**	**Abacavir**
Linearity	
Slope (m)	100.1
Intercept (C)	−26.91
% Y-intercept	−0.01
Correlation coefficient	0.9999
Precision (%RSD)^a^	0.21
Ruggedness(%RSD)^a^	0.37
% Recovery^b^ at…	
50% level	99.6 ± 0.27
100% level	99.7 ± 0.33
150% level	100.0 ± 0.13

a (n=6);

b (n=3).

**Tab. 6.** Evaluation of accuracy for related impurities

**Amount spiked^a^**	**% Recovery^b^**						
	**Imp-A**	**Imp-B**	**Imp-C**	**Imp-D**	**Imp-E**	**Imp-F**	**Imp-G**
LOQ	98.0 ± 0.18	103.0 ± 0.32	96.9 ± 0.47	99.9 ± 0.91	94.5 ± 0.42	98.3 ± 0.12	101.8 ± 0.19
50%	96.7 ± 0.27	101.9 ± 0.81	101.3 ± 0.07	100.7 ± 0.17	99.8 ± 0.49	101.7 ± 0.09	103.1 ± 0.14
100%	103.5 ± 0.33	102.2 ± 0.19	99.9 ± 0.21	98.2 ± 0.95	104.2 ± 0.87	102.1 ± 0.96	100.9 ± 0.13
150%	98.9 ± 0.13	99.3 ± 0.16	97.5 ± 0.45	98.6 ± 0.02	98.6 ± 0.29	100.6 ± 0.19	100.0 ± 0.82

a Amount of impurities spiked with respect to specification level (0.10% for Imp-A, Imp-B, Imp-C, Imp-D, Imp-E, Imp-F and Imp-G with respect to analyte concentration) in analyte solution;

b (n=3)

**Tab. 7.** Results of robustness parameter

**Parameter**	**Actual value**	**Changed value**	**No. of theoretical plates (USP tangent method)**	**USP tailing factor (Tf)**	**Resolution between Abacavir and Imp-C**
Flow rate	0.4 mL/min	0.3 mL/min	72,321	1.1	4.3
		0.5 mL/min	89,546	1.0	2.4

Wave length	220 nm	218 nm	86,781	1.0	3.2
		222 nm	85,453	1.0	3.3

Temperature	40°C	35°C	79,264	1.1	3.4
		45°C	85,946	1.0	3.9

Gradient^a^	0/8, 5/40, 6/40, 6.01/8	0/6, 5/38, 6/38, 6.01/8	69,324	1.1	3.5
		0/10, 5/42, 6/10, 6.01/10	87,951	1.0	2.4

a Time in min/%B.
